# The *Alternaria alternata* StuA transcription factor interacting with the pH-responsive regulator PacC for the biosynthesis of host-selective toxin and virulence in citrus

**DOI:** 10.1128/spectrum.02335-23

**Published:** 2023-10-09

**Authors:** Yanan Chen, Yingzi Cao, Chen Jiao, Xuepeng Sun, Yunpeng Gai, Zengrong Zhu, Hongye Li

**Affiliations:** 1 The Key Laboratory of Molecular Biology of Crop Pathogens and Insects of Ministry of Agriculture, The Key Laboratory of Biology of Crop Pathogens and Insects of Zhejiang Province, Institute of Biotechnology, Zhejiang University, Hangzhou, China; 2 Collaborative Innovation Center for Efficient and Green Production of Agriculture in Mountainous Areas of Zhejiang Province, College of Horticulture Science, Zhejiang Agriculture & Forestry University, Hangzhou, China; 3 Hainan Institute, Zhejiang University, Sanya, China; South China Agricultural University Integrative Microbiology Research Centre, Guangzhou, China

**Keywords:** StuA, PacC, ACT, virulence, *A. alternata*

## Abstract

The tangerine pathotype of *Alternaria alternata* produces a host-selective toxin termed *Alternaria citri* toxin (ACT). The molecular mechanisms underlying the global regulation and biosynthesis of ACT remain unknown. In the present study, the function of an APSES transcription factor StuA was investigated. StuA was shown to be required for ACT biosynthesis and fungal virulence. StuA was found, for the first time, to physically interact with a pH-responsive transcription regulator PacC using yeast two-hybrid, bimolecular fluorescence complementation, and GST pull-down assays. Functional analyses revealed that StuA and PacC regulate the expression of genes involved in toxin biosynthesis and virulence. Mutation of *stuA* via targeted gene deletion or silencing *pacC* yielded fungal strains that decreased the expression of seven toxin biosynthetic genes (*ACCT*) and toxin production. EMSA analyses revealed that PacC could bind to the promoters of *ACTT6* encoding an enoyl-CoA hydratase and *ACTTR* encoding an ACT pathway-specific transcription factor. Site-directed mutagenesis of five potential protein kinase A (PKA) phosphorylation sites in StuA revealed that none of the sites was involved in ACT production, indicating that the function of StuA in the regulation of *ACT* gene expression is not dependent on phosphorylation. Overall, our results confirmed that PacC is one of the key regulators interacting with StuA for the biosynthesis of ACT. Environmental pH may play a decisive role during *A. alternata* pathogenesis. Our results also revealed a previously unrecognized (StuA-PacC)→ACTTR module for the biosynthesis of ACT in *A. alternata*.

**IMPORTANCE:**

In this study, we used *Alternaria alternata* as a biological model to report the role of StuA in phytopathogenic fungi. Our findings indicated that StuA is required for *Alternaria citri* toxin (ACT) biosynthesis and fungal virulence. In addition, StuA physically interacts with PacC. Disruption of *stuA* or *pacC* led to decreased expression of seven toxin biosynthetic genes (ACCT) and toxin production. PacC could recognize and bind to the promoter regions of *ACTT6* and *ACTTR*. Our results revealed a previously unrecognized (StuA-PacC)→ACTTR module for the biosynthesis of ACT in *A. alternata*, which also provides a framework for the study of StuA in other fungi.

## INTRODUCTION

The necrotrophic fungal pathogen *Alternaria alternata* (Fr.) Keissler causes diseases in more than 100 plant species, including many crops and fruits (1, 2). *A. alternata* has several pathogenic variants known as pathotypes. Each pathotype produces a unique host selective toxin (HST) and causes disease in specific host plants (3, 4). The tangerine pathotype of *A. alternata* causes Alternaria brown spot in many susceptible citrus cultivars, including tangerines, grapefruit, and various hybrids of tangerines and citrus (5). If not controlled, the disease could cause a serious problem in citrus production. The tangerine pathotype produces and secretes an HST named *Alternaria citri* toxin (ACT). ACT is extremely toxic to susceptible citrus cultivars. It has been demonstrated that ACT at a concentration as low as 2 × 10^−8^ M can kill citrus cells of susceptible cultivars. In contrast, resistant cultivars can tolerate ACT up to 2 × 10^−4^ M (5 – 7). ACT causes rapid electrolyte leakage and cell death in susceptible citrus cultivars (8, 9). The ability to produce ACT is absolutely required for *A. alternata* pathogenesis because fungal mutants carrying the deletion in genes required for the biosynthesis of ACT fail to cause any symptoms (10).

ACT is a low-molecular-weight secondary metabolite containing three moieties, 9,10-epoxy-8-hydroxy-9-methyl-decatrienoic acid (EDA), a valine, and a polyketide (4). The *ACT* gene cluster consisting of 25 genes is located on a small, conditionally dispensable chromosome (11 – 13). Of them, nine genes have been functionally characterized to be required for the biosynthesis of ACT. Five genes, *ACTT1* encoding an acyl-CoA ligase, *ACTT2* encoding a hydrolase, both *ACTT3* and *ACTT6* encoding an enoyl-CoA hydratase and *ACTT5* encoding an acyl-CoA synthetase are involved in the biosynthesis of the EDA moiety (14 – 16). Two genes, *ACTTS2* encoding an enoyl-reductase and *ACTTS3* encoding a polyketide synthase, are responsible for the elongation of the polyketide chain (17). *ACTS4* encoding a nonribosomal peptide synthetase has recently been demonstrated to be involved in the biosynthesis of ACT in the tangerine pathotype (18). A Zn2Cys6 transcription factor encoded by *ACTTR* is a pathway-specific regulator required for the regulation of the genes in the cluster (18).

The biosynthesis of fungal secondary metabolites is regulated or impacted by a wide variety of proteins, signals, and environmental factors (19, 20). Studies in the tangerine pathotype have identified components in the mitogen-activated protein (MAP) kinase pathways, peroxisome complex activities, and autophagy-related processes that are required for ACT biosynthesis and virulence (21 – 24). A basal transcription factor II H subunit (tfb5) and a GATA transcription factor (AreA) have also been shown to be required for ACT biosynthesis, sporulation, and virulence in the tangerine pathotype (25, 26). However, how those proteins and components are coordinated to form a global network regulating the biosynthesis of ACT remains elusive.

Transcription factors belonging to the family of APSES (Asm1p, Phd1p, Sok2p, Efg1p, and StuA) contain a basic helix-loop-helix (bHLH) DNA binding domain capable of binding to the specific stress response element (STRE) with the consensus sequence (A/T)CGCG(T/A)N(A/C) (27, 28). APSES often forming homo- or heterodimers (29, 30) play diverse biological functions including development, stress responses, and biosynthesis of secondary metabolites in fungi (31, 32). The function of StuA homologs has been studied in some pathogenic fungi with a hemibiotrophic lifestyle. A MoStu1 homolog of *Magnaporthe oryzae* contributes to conidiation, mycelial growth, and appressorium-mediated infection in rice (33). StuA of *Aspergillus fumigatus* is required for the biosynthesis of secondary metabolites (34, 35). StuA of *Ustilago maydis* plays key roles in dimorphism, virulence, and sporulation (36, 37). StuA of *F. graminearum* is required for life cycle transitions and virulence (28). The biological function of StuA remains unknown in fungi with a necrotrophic lifestyle. In the present study, we found that the *A. alternata* StuA physically interacted with a pH-responsive transcription factor (PacC) for the biosynthesis of ACT and fungal virulence.

## RESULTS

### Identification of *stuA* and construction of *stuA* mutants in *A. alternata*

The *stuA* homolog was identified at the AALT_10904 locus from the *A. alternata* genome database (GCA 001572055.1) using the *Aspergillus nidulans* StuA amino acid sequences as a query. *stuA* has an open reading frame of 1,893 bp encoding a putative polypeptide of 631 amino acids. StuA possesses an APSES-type DNA binding domain similar to the other StuA transcription factors of pathogenic fungi (Fig. S1a). Phylogenetic analysis revealed that StuA is the most related to the *Exserohilum turcica* EtStuA (Accession no. XP_008027222.1), sharing 82% sequence similarity (Fig. S1). *stuA* was deleted in the wild-type Z7 strain using homologous recombination, and the deletion transformants were identified by PCR and Southern blot analysis (Fig. S2a through S2c). A complementation strain designated Δ*stuA*-C was generated by introducing the wild-type *stuA* gene with its native promoter into protoplasts prepared from Δ*stuA*.

### *stuA* is required for filamentous growth and virulence

**Fig 1:**
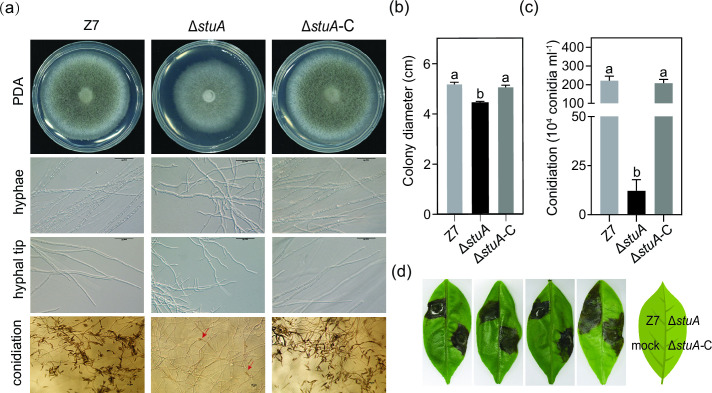
StuA regulates vegetative growth, sporulation, and pathogenicity in *A. alternata*. (**A**) Vegetative growth, hyphal morphology, hyphal tip growth, and sporulation of Δ*stuA*, Z7, and the complementation Δ*stuA*-C strains on PDA. Conidia produced by the Δ*stuA* strain are indicated by red arrows. (**B**) Quantification of colony diameter. Error bars represent standard deviations. Different letters indicate a statistical significance according to the one-way ANOVA test (*P* < 0.05). (**C**) Quantification of conidia production. (**D**) Inoculation of Z7, Δ*stuA,* and Δs*tuA*-C by placing mycelial plugs on detached Hongjv leaves. A blank agar plug was used as the mock. Necrotic lesions were recorded 3 days post-inoculation (dpi).

After being grown on potato dextrose agar (PDA) for 3 days, both wild type and Δ*stuA*-C produced grayish colonies. In contrast, Δ*stuA* produced whitish colonies and reduced growth by 15% compared to the wild type (Fig. 1A and B). Microscopic examination revealed that Δ*stuA* hyphae were stubby and swollen with frequent branching, but hyphae of Δ*stuA*-C and Z7 grew relatively straight with few branching (Fig. 1A). Δ*stuA* produced significantly fewer conidia than Z7 and Δ*stuA*-C, reducing by 94% after a 7-day incubation (Fig. 1A and C). Infection assays by placing mycelial plugs on detached leaves of a susceptible citrus cultivar Hongjv revealed that necrosis lesions induced by Z7 or Δ*stuA*-C developed at the inoculation point and spread rapidly 3 days post-inoculation (dpi). No necrosis was observed on leaves inoculated with Δ*stuA* 3 dpi (Fig. 1D).

### *stuA* is required for toxin biosynthesis

**Fig 2:**
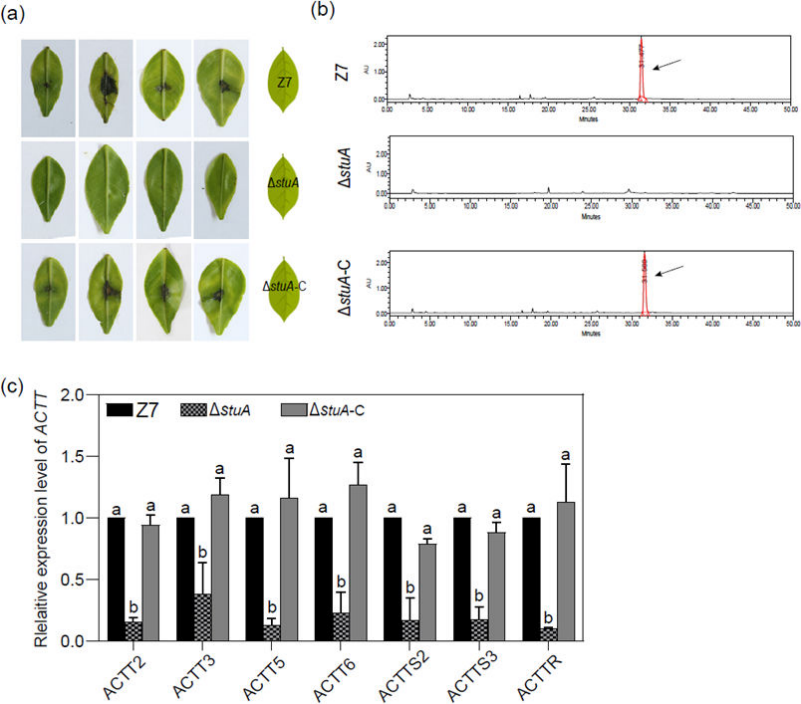
StuA plays a vital role in ACT biosynthesis of *A. alternata*. (**A**) Detached Hongjv leaves were inoculated with 10 µL sterile culture filtrates of each strain to test the toxicity of ACT. Leaves were kept in a plastic box for lesion development. Necrotic lesions were recorded at 6 dpi. (**B**) HPLC analysis of ACT toxin purified from culture filtrates of each strain. The peak representing ACT toxin is indicated by a black arrow. (**C**) The relative expression level of the ACT biosynthetic genes in the Z7 and the Δ*stuA* strains. The actin gene was used as an internal control. Expression of each of the *ACCT* genes in Z7 was set at 1 and used for statistical analysis to determine the relative expression of each gene in Δ*stuA*. Error bars represent standard deviations from three biological replicates. Different letters represent statistical significance according to the one-way ANOVA test (*P* < 0.05).

A leaf necrotic bioassay conducted on detached leaves (*Citrus reticulata* Blanco, cv. Hongjv) revealed that samples purified from culture filtrates of Δ*stuA*-C and Z7 resulted in dark brown necrotic lesions 3 days after treatment (Fig. 2A). However, samples prepared from culture filtrates of Δ*stuA* failed to induce visible lesions. HPLC analyses of samples purified from culture filtrates of Δ*stuA*-C and Z7 identified a peak with a retention time of 32 min, which was completely absent in samples prepared from culture filtrates of Δ*stuA* (Fig. 2B). Analyses of RNA samples by quantitative RT-PCR revealed that the transcript levels of *ACTT2*, *ACTT3*, *ACTT5*, *ACTT6*, *ACTTS2*, *ACTTS3*, and *ACTTR* were significantly downregulated in Δ*stuA* compared with the Z7 strain (Fig. 2C). The expression levels in Δ*stuA*-C were not significantly different from the Z7 strain.

### Protein kinase A phosphorylation sites in StuA have no impact on ACT biosynthesis

Computer prediction identified five putative PKA phosphorylation sites, serine (S67, S370, and S411) and Threonine (T110 and T601) in StuA (Table S2). Each of the sites was changed to alanine (A) in pNEO1300-StuA by site-directed mutagenesis. The resultant plasmid was introduced into protoplasts of Δ*stuA* to generate five fungal strains Δ*stuA*-C^S67A^, Δ*stuA*-C^T110A^, Δ*stuA*-C^S370A^, Δ*stuA*-C^S411A^, and Δ*stuA*-C^T601A^. All strains except Δ*stuA*-C^T110A^ displayed wild-type morphology (Fig. S3a) and virulence (Fig. S3b). Δ*stuA*-C^T110A^ produced a whitish colony and reduced virulence. However, HPLC analyses revealed that Δ*stuA*-C^T110A^ and wild type produced similar levels of ACT (Fig. S3c). The Δ*stuA*::StuA-GFP strain produced green fluorescent spots resembling that of 4′,6-diamidino-2-phenylindole (DAPI) staining, and the Δ*stuA*::StuA^T110A^-GFP strain produced green fluorescence in both the nucleus and the cytoplasm (Fig. S3d).

### StuA interacts directly with the pH-responsive transcription factor PacC

**Fig 3:**
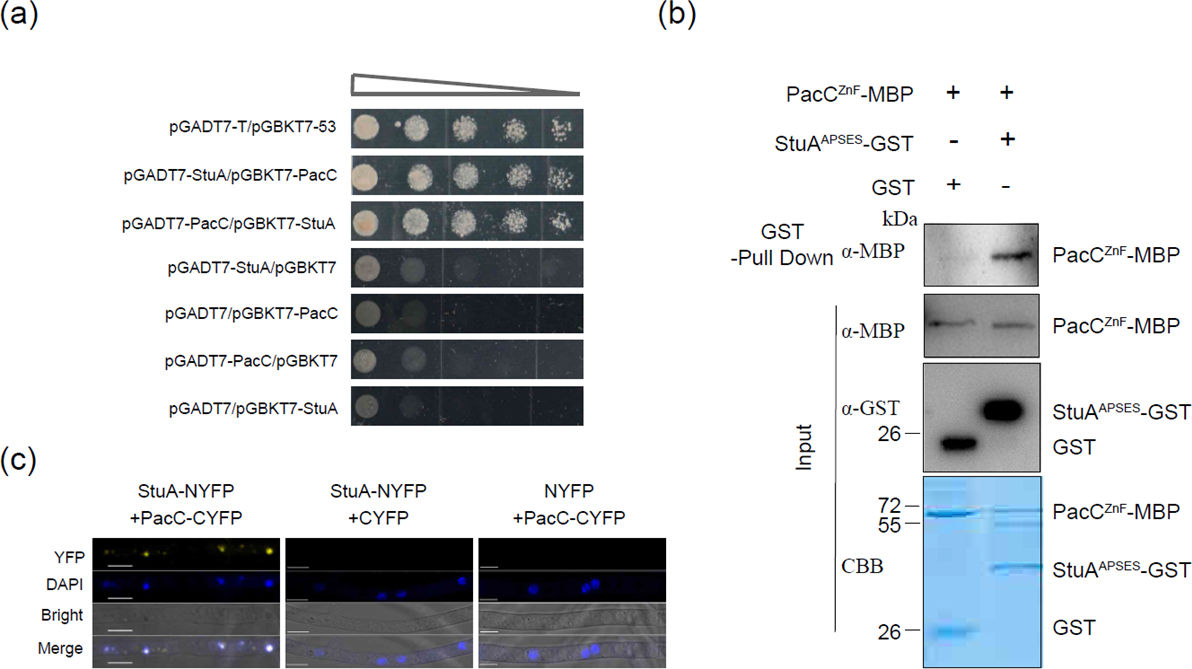
StuA physically interacts with PacC. (**A**) Y2H analysis reveals the interaction of StuA with PacC. Serial dilutions of yeast cells (cells/mL) transferred with the bait and prey constructs indicated in the figure were assayed for growth on SD/−Ade/−His/−Leu/−Trp plates. Pairing pGADT7-StuA (bait) and pgbkt7-PacC (prey) resulted in the growth of the yeast strain on medium without Ade, His, Leu, and Trp. Similar results were obtained in reverse by pairing pGADT7-PacC (bait) and pgbkt7-StuA (prey). Pairing pGBKT7-53 and pGADT7 was used as a positive control (**B**). The APSES domain of StuA (StuA^APSES^) interacted with the ZnF_C2H2 domain of PacC (PacC^ZF^) *in-vitro* by GST pull-down assays. StuA^APSES^ and PacC^ZF^ were fused to GST and MBP tags, respectively. StuA^APSES^-GST or GST-bound resin was incubated with crude protein extracts containing PacC^ZF^-MBP and analyzed by western blot analyses. Proteins were detected by staining with Coomassie Brilliant Blue (CBB). (**C**) The interaction of PacC with StuA in the nucleus was visualized by BiFC assays. YFP signals were observed in vegetative hyphae of the transformant harboring YFPN-StuA and YFPC-PacC. Scale bar = 5 µm.

Searching yeast interactome database (https://thebiogrid.org/) with the yeast SOK2 (a homolog of StuA) as a query identified 248 proteins that potentially interacted with StuA (Table S3). Of those proteins, the yeast Rim101, orthologous to PacC in filamentous fungi, was found to likely interact with SOK2. The *A. alternata* PacC coding sequence was used to construct a vector for yeast two hybridization (Y2H) assays. Mixing Y2H Gold strains carrying pGADT7-StuA (bait) and pGBKT7-PacC (prey) allowed the yeasts to grow on a selective medium without His, Leu, Trp, and Ade (Fig. 3A). Similar results were obtained in reverse by pairing pGADT7-PacC (bait) and pGBKT7-StuA (prey). To further confirm the interaction between StuA and PacC, recombinant PacC^ZF^-MBP, glutathione StuA^APSES^-glutathione S-transferase (GST), and GST (as a negative control) were independently expressed in an *Escherichia coli* BL21 strain. Pull-down assays revealed that PacC^ZF^-MBP could be detected in the StuA^APSES^-GST fraction but not in the GST fraction (Fig. 3B). BiFC assays also revealed that co-transforming StuA-NYFP and PacC-CYFP constructs into the protoplasts of wild type resulted in yellow fluorescent spots resembling that of DAPI staining (Fig. 3C).

### *pacC* is also required for ACT biosynthesis and virulence in *A. alternata*

**Fig 4:**
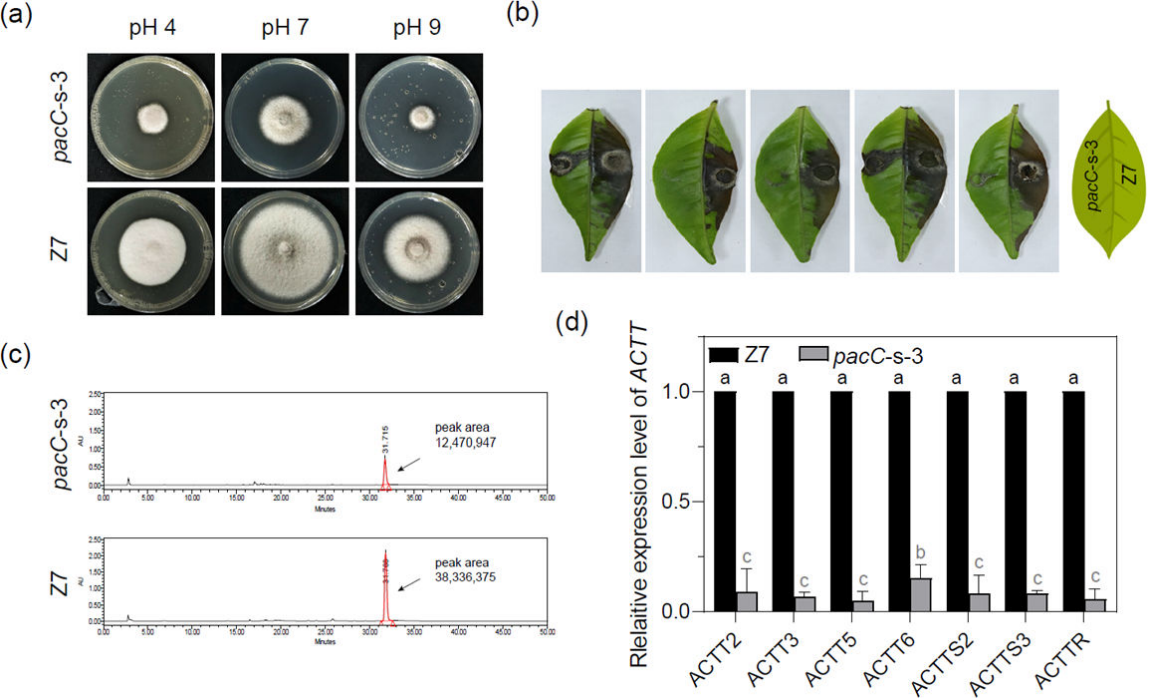
PacC is required for pathogenicity and ACT biosynthesis. (**A**) Growth of the silencing mutant *pacC*-s-3 under different pH conditions compared with Z7. All strains were cultured on CM medium for 3 days and transferred to PDA plates with different pH values (4, 7, or 9). (**B**) Necrotic lesions formed on detached Hongjv leaves by the *pacC* mutants and Z7 strains 3 dpi. (**C**) HPLC analysis of ACT toxin purified from culture filtrates of *pacC*-s-3 and Z7. ACT toxin is indicated by a black arrow. (**D**) The relative expression level of ACT biosynthetic genes in Z7 and *pacC*-s-3 strains. The actin gene was used as an internal control. Expression of each of the *ACCT* genes in Z7 was set at 1 and used for statistical analysis to determine the relative expression of each gene in the *pacC* mutants. Error bars represent standard deviations from three biological replicates. Different letters represent statistical signiﬁcance according to the one-way ANOVA test (*P* < 0.05).

As with all fungal PacC homologs, PacC contains three highly conserved Cys2His2 zinc finger DNA binding domains in *A. alternat*a (Fig. S4a). However, phylogenetic analyses revealed that PacC was distant from other fungal PacC homologs (Fig. S4b). Several attempts to disrupt *pacC* using homologous recombination failed to obtain successful mutants after screening more than 100 transformants by PCR using three different sets of primers. Thus, RNA interference (RNAi) was performed to evaluate the function of *pacC* in *A. alternata* (Fig. S4c). Transforming the pSilent-1-*pacC* construct into protoplasts prepared from the Z7 strain identified three transformants carrying pSilent-1-*pacC*. Quantitative RT-PCR analyses revealed that all transformants reduced the expression levels of *pacC* to varying degrees. One transformant, designated *pacC*-s-3, was found to reduce the expression of *pacC* by 75% compared to the Z7 strain (Fig. S4d). The growth of *pacC*-s-3 was reduced particularly under acidic and alkaline conditions (Fig. 4A). Virulence assays on Hongjv leaves revealed that the *pacC*-s-3 strain induced much smaller necrotic lesions than the wild type (Fig. 4B). Noticeably, some leaf spots inoculated with *pacC*-s-3 showed no necrotic lesions. HPLC analysis revealed that *pacC*-s-3 decreased ACT production by 67% compared to the wild type (Fig. 4C). Quantitative RT-PCR analyses revealed that the expression of *ACTT2*, *ACTT3*, *ACTT5*, *ACTT6*, *ACTTS2*, *ACTTS3*, and *ACTTR* was significantly downregulated in *pacC*-s-3 compared to the Z7 strain (Fig. 4D).

### PacC binds to the promoters of *ACTT6* and *ACTTR*

**Fig 5:**
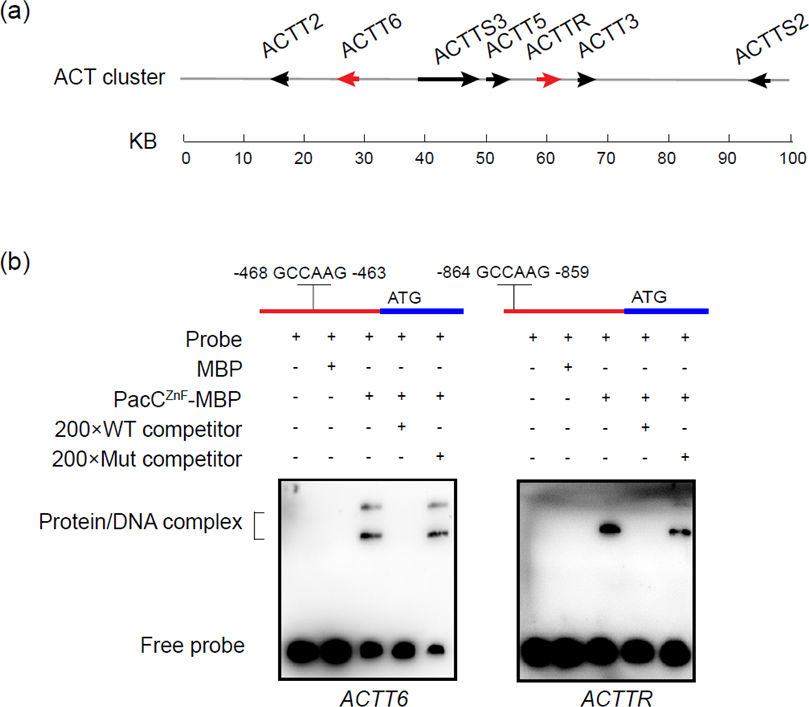
PacC directly regulates transcription of ACT biosynthetic genes. (**A**) Physical position of ACT biosynthetic cluster. Red arrows correspond to the genes having a PacC binding motif 1 kb upstream of the first ATG codon. (**B**) Validation of the interaction between PacC and putative binding motif in the promoter of three ACT biosynthetic genes by electrophoretic mobility shift assay (EMSA). A schematic diagram shows the predicted binding motif of PacC in the promoter region of ACT biosynthetic genes (upper panel). The promoter of each gene was incubated with purified PacC^ZF^-MBP or MBP at 28°C for 20 min.

Analysis of sequences upstream of the putative ATG translational start codon of all 25 *ACT* genes identified a 5′-GCCARG-3′ motif, a putative binding site of PacC, only in the promoter regions of *ACTT6* and *ACTTR* (Fig. 5A). Electrophoretic mobility shift assay (EMSA) using the purified PacC^ZF^-MBP protein (a polypeptide containing a PacC binding domain tagged with MBP) was performed to determine whether or not PacC containing three Cys2His2 zinc finger DNA binding domains could recognize and bind to 5′-GCCARG-3′. Mixing PacC^ZF^-MBP with the 5′ DNA fragments of *ACTT6* and *ACTTR* resulted in a DNA mobility shift (Fig. 5B). Adding an excess of the wild-type unlabeled DNA fragment abolished binding as no DNA mobility shift was detected. However, adding an unlabeled but mutated DNA fragment containing 5′-GAACCG-3′ resulted in a mobility shift.

## DISCUSSION

**Fig 6:**
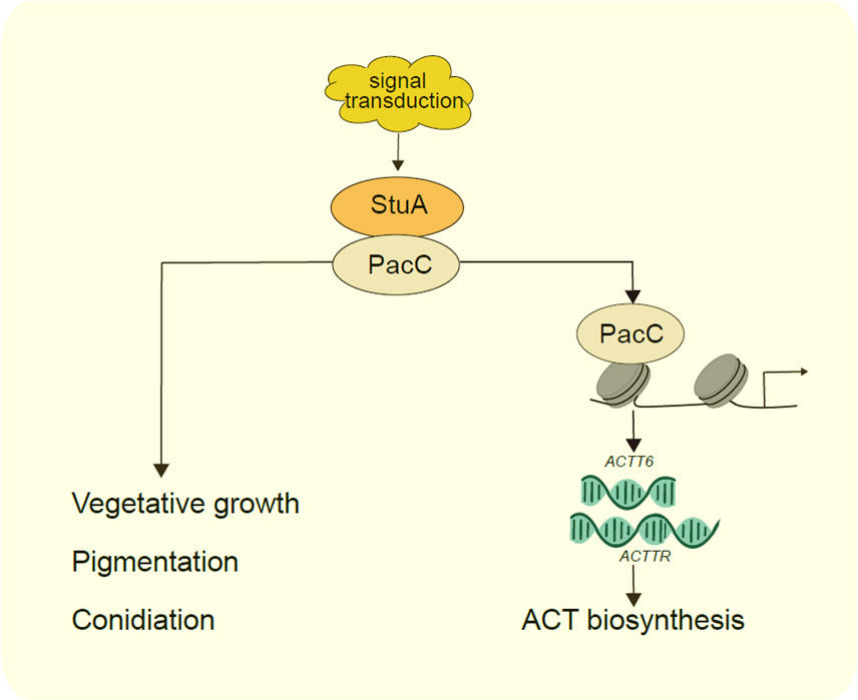
A proposed model for StuA-mediated regulation of ACT biosynthesis. Deleting *stuA* results in severe vegetative growth, sporulation, ACT production, and pathogenicity of *A. alternata*. In addition, StuA interacts with a pH-responsive transcription factor PacC, which increases the transcript levels of the ACT biosynthetic genes *ACTT6* and *ACTTR* by directly binding to its promoter.

APSES transcription factors play important roles in a wide range of biological processes in saprophytic, human pathogenic, and phytopathogenic fungi (31, 35, 38, 39). StuA is the key member of the APSES family. Its function as a transcription factor has been studied in many filamentous fungi; however, its regulatory action on PacC-mediated signaling has never been demonstrated. In the present study, we demonstrated, for the first time, that StuA could interact with the pH-responsive transcription factor PacC (Fig. 6). Furthermore, we provided evidence that PacC affects ACT production and virulence of *A. alternata* by directly regulating the expressions of genes required for ACT biosynthesis. Experiments were also conducted to demonstrate that PacC can physically bind to the promoter regions of *ACTT6* encoding an enoyl-CoA hydratase and *ACTTR* encoding an ACT pathway-specific transcription factor. These findings uncover an important protein-interaction module (StuA-PacC)→ACTTR→ACTT that controls ACT production and virulence in the tangerine pathotype of *A. alternata*.

StuA is required for *A. alternata* growth. Growth reduction has been reported in *stuA* mutants of *F. graminearum* and *Arthrobotrys oligospora* (28, 32). Moreover, deleting *stuA* resulted in a drastic decrease in conidia production. StuA is also required for conidiation in *Magnaporthe grisea*, *F. graminearum*, *Stagonospora nodorum*, and *A. nidulans* (27, 28, 40, 41). The results confirm the important role of StuA in hyphal differentiation and the development of fungi. Although growth and conidiation are impaired in Δ*stuA*, the major deficiency that impacts the Δ*stuA* virulence is its inability to produce ACT and infect citrus plants. ACT is a secondary metabolite, which is released during spore germination and host colonization, and is absolutely required for the virulence of *A. alternata* (4, 42). StuA impacts the biosynthesis of ACT by transcriptionally regulating the expression of ACT biosynthetic genes located on the *ACT* gene cluster. This regulatory function was demonstrated to be mediated via PacC in this current study. Thus, we concluded that StuA is one of the key virulence determinants in the tangerine pathotype of *A. alternata*. Similarly, StuA has been shown to be required for virulence in *S. nodorum* (41), *Leptosphaeria maculans* (43), *U. maydis* (37), and *A. oligospora* (32). However, deleting *stuA* has no impact on the virulence of *F. oxysporum* (44). Those studies indicate that StuA has different pathological functions in different fungi.

The biosynthesis of fungal toxins is controlled by genes commonly found in clusters scattered throughout the genome (45). Global transcription factors responding to environmental stimuli and developmental signals are essential regulators for the biosynthesis of fungal toxins. StuA is one of the key regulators for the biosynthesis of fungal secondary metabolites. For example, the expression of genes involved in the biosynthesis of epipolythiodioxopiperazine (ETP) gliotoxins is StuA-dependent in *A. fumigatus* (34). In *Fusarium verticillioides*, StuA is required for the production of fumonisin and the expression of genes involved in the biosynthesis of fumonisin and fusarin C (46). StuA is involved in the production of a mycotoxin (Alternariol) in *S. nodorum* (41). Despite StuA has been shown to be essential for producing a number of secondary metabolites in filamentous fungi, the underlying mechanism remains elusive. As demonstrated in the current study, StuA is required for the biosynthesis of ACT and virulence. Five potential PKA phosphorylation sites were found in StuA; however, none of the sites impacts ACT production after site-directed mutagenesis. The results indicate that the StuA activity in relation to ACT production is not regulated by cAMP/PKA-induced phosphorylation. Through computer prediction and experimental verification, we have demonstrated that StuA physically interacted with a pH-responsive transcription factor PacC, and the expression level of *pacC* was decreased in the Δ*stuA* strain but not Δ*stuA*-C^T110A^, suggesting that *pacC* can be regulated by StuA and is independent of the T110 phosphorylation site of StuA (Fig. S4d). Functional characterization demonstrated that StuA regulates the production of ACT in *A. alternata* by regulating the ambient pH-responsive regulator *pacC*. The results also indicate that sensing environmental pH is important for gene expression and toxin production.

PacC has been shown to be required for development, toxin production, and virulence in various fungi (47). During the hemibiotrophic stage, the rice blast fungus *M. oryzae* depends on PacC-mediated sensing to manipulate host cellular pH (48). The postharvest pathogenic fungus *Penicillium expansum* secrets ammonia to activate a PacC homolog and increases the accumulation of the mycotoxin patulin (PAT) during fruit colonization (49). PacC positively regulates 15 cluster genes involved in the biosynthesis of PAT in *P. expansum* as all 15 genes are downregulated in a *PacC* knockout mutant (50). The promoter regions of nine PAT cluster genes contain one or more PacC-binding consensus sequences, suggesting that PacC likely affects the biosynthesis of PAT by regulating the PAT gene cluster (50). PacC acts as a transcriptional activator to regulate gene expression by binding to the consensus sequence (5′-GCCARG-3′) (51, 52). In the present study, we have demonstrated that the purified PacC could directly bind to the promoter regions of *ACTT6* and *ACTTR*, which are known to be critical components in the biosynthesis of ACT. Silencing *pacC* resulted in markedly reduced production of ACT, supporting the important role of PacC in the biosynthesis of ACT (10, 18). Given that environment pH is a common stress that *A. alternata* might encounter, especially at the initial stage of infection, it remains unclear how pH stress would affect the PacC-mediated regulation for the production of ACT during fungal colonization.

In summary, this study established a close link between StuA and PacC for the biosynthesis of ACT in *A. alternata*. Both StuA and PacC also are required for growth, conidiation, and virulence. We demonstrated that StuA physically interacts with PacC, which in turn transcriptionally regulates the expression of seven *ACTT* genes required for the biosynthesis of ACT. PacC recognizes and binds to the promoter regions of *ACTT6* and *ACTTR*. No consensus sequence (5′-GCCARG-3′) in the promoter region of other *ACT* genes, indicating that PacC regulates the pathway-specific regulator ACTTR, which in turn regulates the expression of other genes in the ACT biosynthetic gene cluster. Because the APSES transcription factors usually regulate downstream genes through the formation of homo- or heterodimers (29, 30), further investigation will allow us to identify other components that interact with StuA and also play a key role in the biosynthesis of ACT.

## MATERIALS AND METHODS

### Fungal strains and culture conditions

The wild-type Z7 strain of *A. alternata* (deposited at China General Microbiological Culture Collection Center under the accession number CGMCC3.18907) was isolated from an infected citrus (Ougan) leaf in Zhejiang, China (53, 54). Unless otherwise indicated, fungal strains were grown on PDA (Solarbio, Beijing, China) at 26°C. Conidia were harvested from fungal cultures grown on PDA for 8 days. PDA powder was buffered with 0.2 M Na_2_HPO_4_ and 0.1 M citric acid to pH 4.0, 7.0, or 9.0 and used in the pH shift experiments. Mycelium was obtained from 36-h liquid cultures at 26°C for DNA, RNA, and protein extraction as well as microscopic observation. Fungal strains were grown in a flask with 300-mL Richard’s solution at 26°C for 7 days to induce the production of ACT toxin.

### Gene deletion and complementation

Gene deletion mutants were generated using fungal transformation system described elsewhere (10). Briefly, a 2-kb upstream flanking sequence fragment and a 2-kb downstream flanking sequence were amplified with the primer pairs stuA-up-F/stuA-up(hph)-R and stuA-down(hph)-F/stuA-down-R, respectively, from Z7 genomic DNA. The two fragments were joined together with the hygromycin resistance gene cassette (HPH) by overlapping PCR to produce two split HPH fragments. The fused fragments were purified and transformed into Z7 protoplasts. The protoplast-mediated transformation was carried out following the protocol described by Lin and Chung (55). Transformants were selected on PDA supplemented with 100 µg/mL hygromycin (Roche Applied Science, Indianapolis, IN, USA) and verified by PCR and Southern blot hybridization. For complementation, a 3,625-bp DNA fragment including the 5′ untranslated region (1.5 kb) and the full-length *stuA* gene was amplified with the primer pair (neo1300) *stuA*-F/*stuA* (neo1300)-R and cloned into the plasmid pNeo1300 containing a G418-resistance gene to yield pNeo1300-*stuA*. The plasmid was transformed into protoplasts prepared from a Δ*stuA*. Putative transformants were examined by PCR and validated further by Southern blot analysis. All primers used in this study are listed in Table S1.

### RNAi vector construction and transformation

Two 300-bp *pacC* fragments were amplified with the primer pairs Psilence-pacC-LF/Psilence-pacC-LR (for sense fragment) and Psilence-pacC-RF/Psilence-pacC-RR (for antisense fragment) from the cDNA library of *A. alternata*. The fragments were cloned into a pSilent-1 vector following the homologous recombination ligation method (56) to yield an RNAi pSilent-1-*pacC* vector containing the hygromycin-resistance gene.

### Site-directed mutagenesis

The S67, T110, S370, S411, and T601 residues of StuA were independently substituted by alanine according to the manual of ClonExpress Ultra One Step Cloning Kit (Vazyme, Nanjing, China) using the pNEO1300-StuA plasmid as the template and the respective primer pairs. Point mutations were confirmed by sequencing using the primers listed in Table S1.

### Virulence assays and HPLC analysis

Virulence assays were performed by placing 5-mm mycelia plugs on 7–15 days old detached healthy Hongjv leaves and kept in a moist plastic box at 26°C for 2 or 4 days. For toxicity assays, fungal strains were cultured in a 300-mL Richard’s solution at 26°C for 28 days on a rotary shaker set at 60 rpm. The pH of culture filtrates was adjusted to 5.5 using 10% phosphate buffer (NaH_2_PO_4_,) and the solution was mixed with 10 mL Amberlite XAD-2 resin with magnetic stirring for 2 h. The resin was filtered with filter paper, and the crude ACT extract was eluted with 40 mL methanol. About 10 μL of crude extracts were placed on the detached Hongjv leaves, and the leaves were kept in a plastic box for 3 or 5 days. All tests were done at least three times, each with three replicates.

ACT toxin was analyzed by high-performance liquid chromatography (HPLC) analysis as previously described. In brief, the 40 mL toxin crude extract was concentrated to 1 mL in a rotary evaporator. ACT was separated by an XbridgeTM18.5 column (4.6 × 250 mm^2^) connected to the Waters 880-PU HPLC system (Japan Spectroscopic, Tokyo, Japan) using methanol/0.1% acetic acid gradient solvent system at a flow rate of 1 mL/min. ACT was detected by the absorbance value at 290 nm. Extracts with a peak retention time of 30–35 min were collected and used for bioactivity analysis. Richard’s medium with blank PDA plugs was extracted in a similar manner and used as a negative control.

### Protein-protein interactions

For yeast two-hybrid screening (Y2H), the coding sequence of each gene was amplified from the cDNA of Z7 with primer pairs listed in Table S1. Full-length cDNA fragments were independently cloned into the vector pGADT7 (bait) or pgbkt7 (prey). The resultant clones were co-transformed into the *Saccharomyces cerevisiae* Y2H Gold strain. Plasmids were sequenced to ensure error-free. The plasmids pGBKT7-53 and pGADT7-T were used as the positive control. Transformants were grown at 30°C for 3 days on a synthetic medium (SD) lacking Leu and Trp, serially diluted, and transferred to SD without His, Leu, Trp, and Ade to assess binding activity. Three independent experiments were performed for each Y2H assay.

For GST pull-down assays, cDNA containing the StuA^APSES^ domain was cloned into pGEX-4T to generate GST-tagged StuA^APSES^-GST protein, and cDNA containing PacC^ZF^ was cloned into PMAL-c5x to generate an Mbp-tagged PacC^ZF^-MBP protein. To test *in-vitro* binding between MBP- and GST-tagged proteins, the GST-tagged protein or GST (negative control) was mixed with glutathione beads, incubated at 4°C for 1 h, mixed with the MPB-tagged protein, and incubated for an additional 3 h with shaking. The beads were washed five times with phosphate-buffered saline, and GST pull-down proteins were examined by western blot analysis using the monoclonal mouse Anti-GST (EM08071, HuaAn bBiotech, Hangzhou, China, 1:5,000 dilution) and the monoclonal mouse anti-MBP antibody (AE016, ABclonal, Wuhan, China, 1:1,000) antibodies. The experiment was repeated three times. BiFC analysis was performed as previously described (57). The fusion constructs were generated by cloning the corresponding cDNA fragments into PKD2-YFPN and PKD5-YFPC vectors. Plasmids YFPN and YFPC were co-transformed into the wild-type strain, and transformants were screened for resistance to both hygromycin and chlorimuron. The resulting transformants were analyzed by PCR. Epifluorescence microscopy was performed using a Zeiss LSM780 confocal microscope (Gottingen, Niedersachsen, Germany).

### Microscopy

Fungal hyphae were examined with a Nikon microscope equipped with an LV100ND image system (Nikon, Japan). For the proteins tagged with YFP, each strain was cultured in PDB on a shaker set at 160 rpm, 26°C for 36 h. The confocal microscopy images were obtained using LSM780 (Gottingen). Fungal nuclei were stained with 1 mg/mL DAPI (Sigma, St. Louis, MO, USA). Each experiment was repeated three times.

### RNA extraction and quantitative RT-PCR

Fungal strains were grown on CM agar medium for 3 days, transferred to Richard’s medium for 7 days, and mycelium was harvested for RNA isolation. Each strain had three biological replicates. Total RNA was extracted from the mycelia of each sample with TRIzol (Takara, Biotechnology, Dalian, China), and reverse transcription was performed with a HiScript II 1st Strand cDNA Synthesis Kit (Vazyme Biotech, Nanjing, China). The relative expression level of each gene was determined by quantitative RT-PCR with HiScript II Q RT SuperMix (Vazyme Biotech). The expression of the actin gene was included as a reference. The experiment was repeated three times.

### Electrophoretic mobility shift assay

EMSA analysis was performed using a LightShift Chemiluminescent EMSA Kit 20148 (Thermo Fisher, USA). The cDNA encoding the StuA^APSES^ domain was amplified and cloned into pGEX-4T and PMAL-c5x vector to generate a GST-tagged protein and an Mbp-tagged protein, respectively. The resulting constructs were individually transformed into the *E. coli* strain BL21 (DE3) and purified for the verification of the cDNA sequence. The recombinant protein PacC^ZnF_C2H2^-MBP was generated as described above. DNA probes were prepared by labeling biotin at the 3̍′-end with annealing complementary oligonucleotides. The purified MBP was used as a negative control. Protein-DNA complexes were separated on 6% native polyacrylamide gels in 0.5 × TBE and transferred to a positively charged nylon membrane (Millipore, Burlington, MA, USA). Biotin-labeled probes were detected according to the instructions of the manufacturer.

### Statistical analysis

Data analyses and plotting were performed using SPSS Statistics 19 (IBM, New York, USA) and GraphPad Prism 9.0.0 (GraphPad Software). Means indicated by the same letters are not significantly different from one another, *P* < 0.05.

## Data Availability

The data that support the findings of this study are available from the corresponding author upon reasonable request.

## SUPPLEMENTAL MATERIAL

Fig. S1 spectrum.02335-23-s0001.pdf Sequence analysis of StuA in A. alternata.

Fig. S2 spectrum.02335-23-s0002.pdf Validation of ΔstuA and ΔstuA-C.

Fig. S3 spectrum.02335-23-s0003.pdf Site-directed mutagenesis analysis with the StuA protein.

Fig. S4 spectrum.02335-23-s0004.pdf Verification of pacC mutants.

Fig. S5 spectrum.02335-23-s0005.pdf The relative area of necrotic lesions caused by A. alternata strains to inoculated leaves.

Supplemental legends spectrum.02335-23-s0006.docx Legends of Fig. S1 to S5 and Tables S1 to S3.

Table S1 spectrum.02335-23-s0007.xlsx Oligonucleotide primers used in this study.

Table S2 spectrum.02335-23-s0008.pdf Putative PKA phosphorylation sites of AaStuA.

Table S3 spectrum.02335-23-s0009.xlsx Proteins putatively interacting with SOK2 in S. cerevisiae.

ASM does not own the copyrights to Supplemental Material that may be linked to, or accessed through, an article. The authors have granted ASM a non-exclusive, world-wide license to publish the Supplemental Material files. Please contact the corresponding author directly for reuse.
