# Supplementary figures and images for: The Alternaria alternata StuA transcription factor interacting with the pH-responsive regulator PacC for the biosynthesis of host-selective toxin and virulence in citrus
Source: Microbiol Spectr. 2023 Oct 9;11(6):e02335-23. doi: 10.1128/spectrum.02335-23 (PMC10715145; doi:10.1128/spectrum.02335-23)

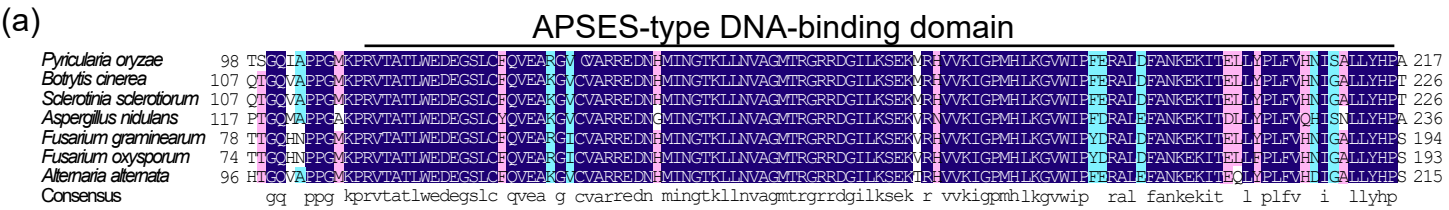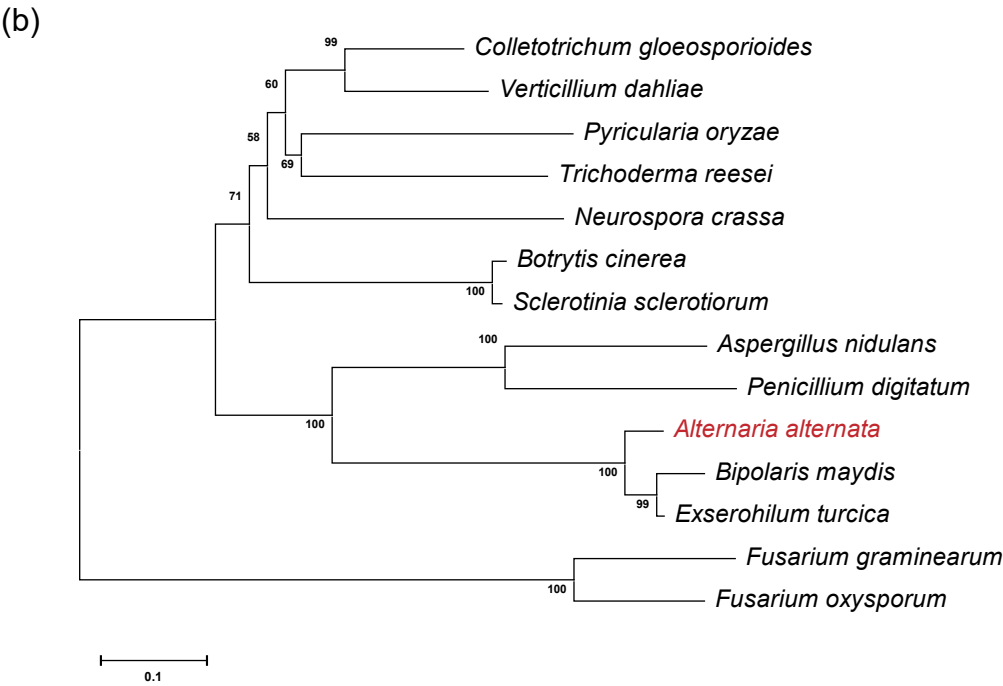

Supplement: Fig. S1 — Sequence analysis of StuA in A. alternata. [file spectrum.02335-23-s0001.pdf]

(a)

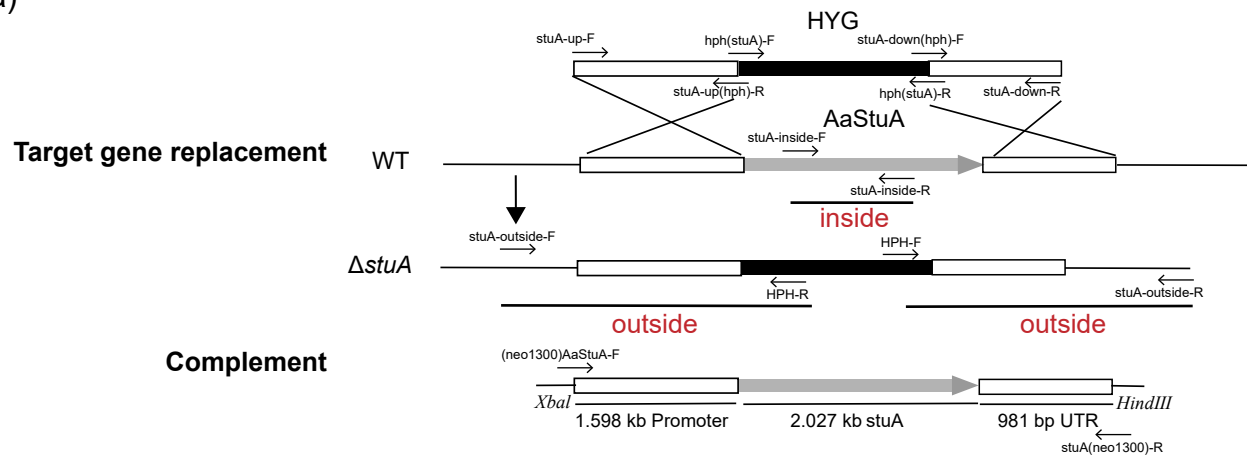

(b)

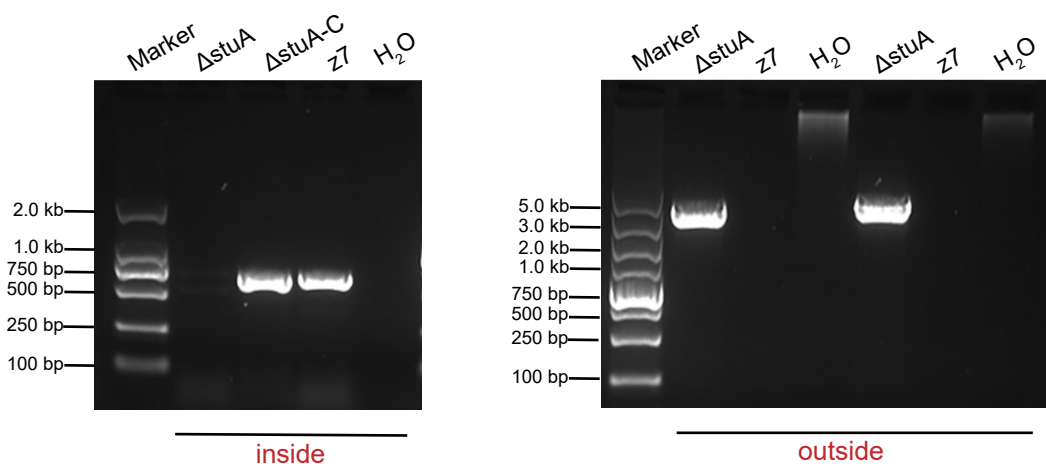

(c)

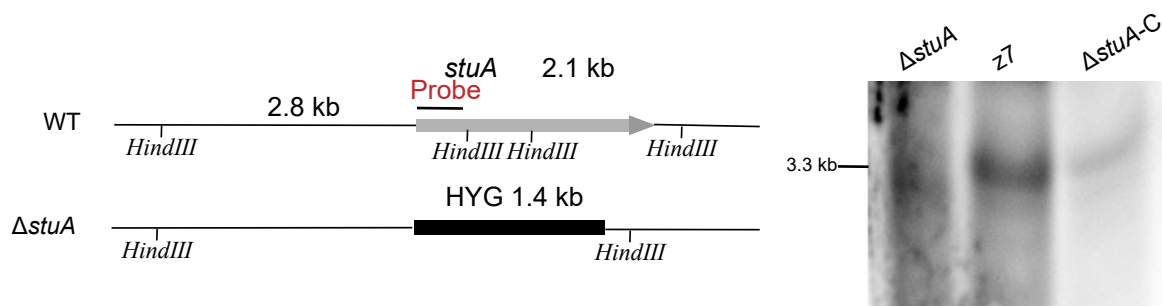

Supplement: Fig. S2 — Validation of ΔstuA and ΔstuA-C. [file spectrum.02335-23-s0002.pdf]

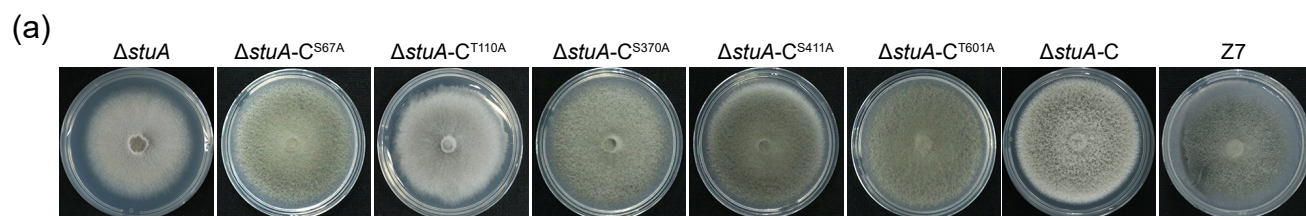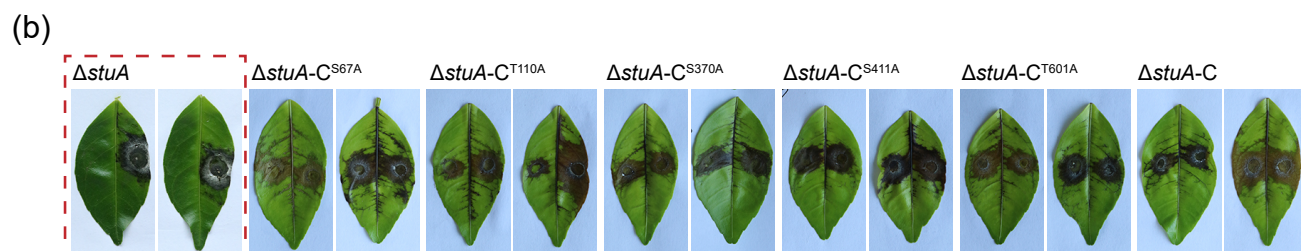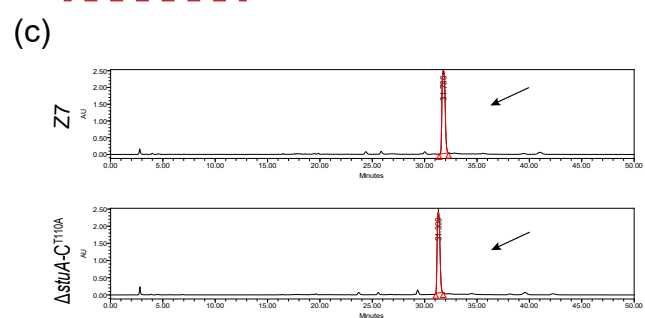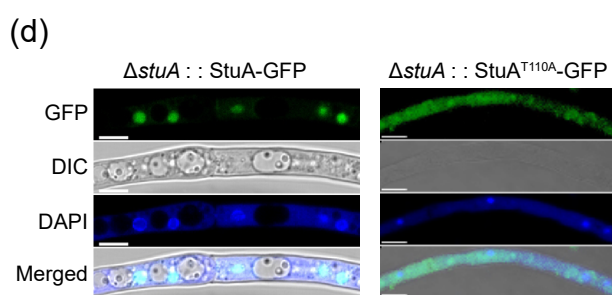

Supplement: Fig. S3 — Site-directed mutagenesis analysis with the StuA protein. [file spectrum.02335-23-s0003.pdf]

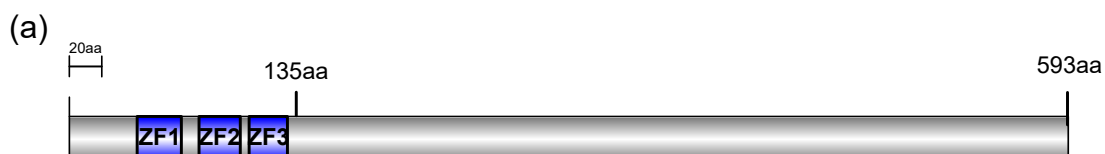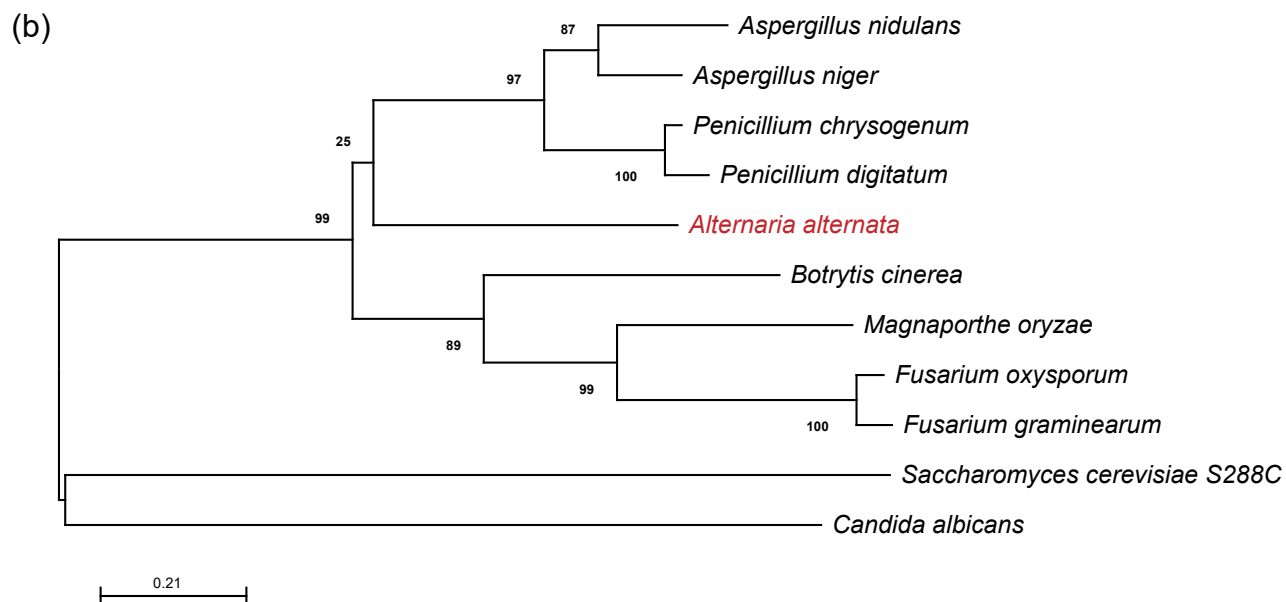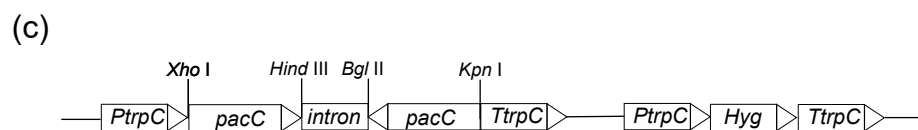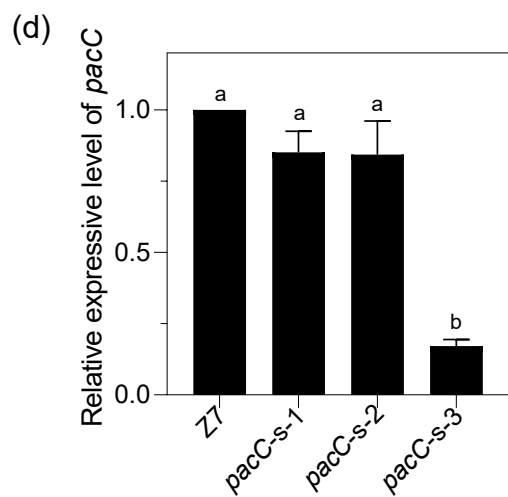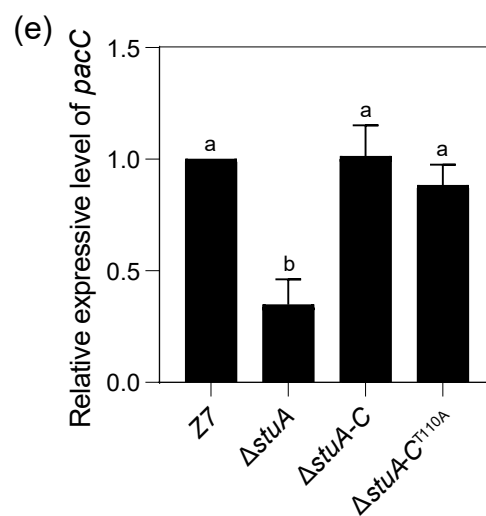

Supplement: Fig. S4 — Verification of pacC mutants. [file spectrum.02335-23-s0004.pdf]

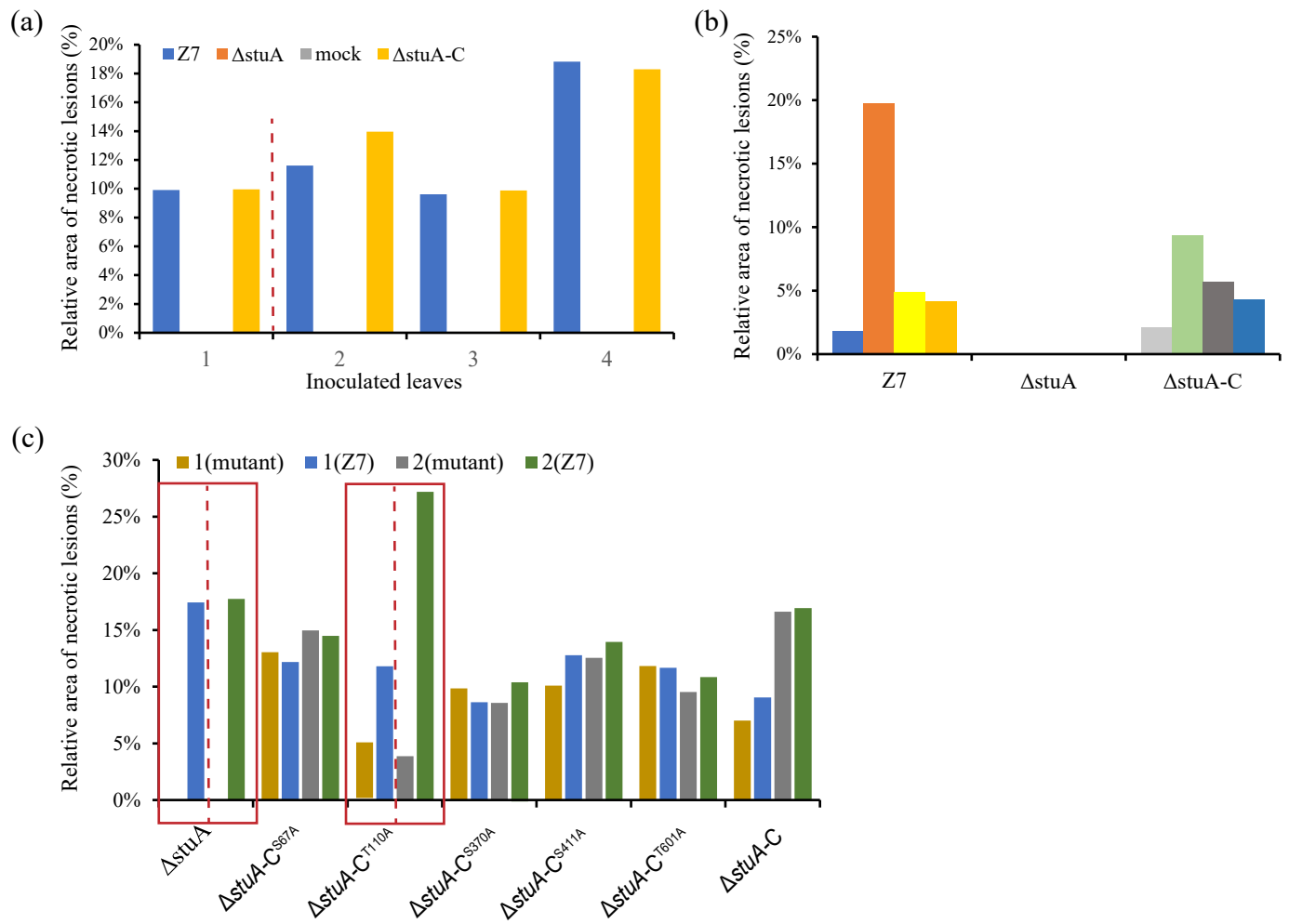

Supplement: Fig. S5 — The relative area of necrotic lesions caused by A. alternata strains to inoculated leaves. [file spectrum.02335-23-s0005.pdf]
