# Supplementary material for: The Alternaria alternata StuA transcription factor interacting with the pH-responsive regulator PacC for the biosynthesis of host-selective toxin and virulence in citrus
Source: Microbiol Spectr. 2023 Oct 9;11(6):e02335-23. doi: 10.1128/spectrum.02335-23 (PMC10715145; doi:10.1128/spectrum.02335-23)
Supplement: Supplemental legends — Legends of Fig. S1 to S5 and Tables S1 to S3. [file spectrum.02335-23-s0006.docx]

**Fig. S1 Sequence analysis of StuA in *A. alternata***. **(a)** Multiple sequence alignment of StuA and its homologues. An APSES-type DNA-binding domain is marked with black lines. The sequences are from the following species: *Pyricularia oryzae* (XP_003718315), *Botrytis cinerea* (XP_024548075), *Sclerotinia sclerotiorum* (APA12740), *A. nidulans* (XP_663440), *F. graminearum* (XP_011319067), F*. oxysporum* (XP_018240414), *A. alternata* (AALT_10904). **(b)** Phylogenetic analysis of StuA in *A. alternata* (indicated with red). The homologues including *Colletotrichum gloeosporioides* (EQB44380), *Verticillium dahlia* (XP_009651260), *P. oryzae* (XP_003718315), *Trichoderma reesei* (XP_006961492), *Neurospora crassa* (XP_960837), *B. cinerea* (XP_024548075), *S. sclerotiorum* (APA12740), *A. nidulans* (XP_663440), *Penicillium digitatum* (EKV13321), *Bipolaris maydis* (XP_014074428), *F. graminearum* (XP_011319067), F*. oxysporum* (XP_018240414). The phylogenetic tree was reconstructed using a maximum likelihood (ML) method after alignment by the CLUSTALW algorithm using the MEGAX program. The horizontal bar indicates the relative distance in the phylogenetic tree.

**Fig. S2** **Validation** **of Δ*stuA* and Δ*stuA-*C.** **(a)** Strategies used for gene knockout and genetic complementation in this study. The target region was replaced with a fragment containing the hygromycin B-resistance cassette (HPH) to create the mutant. **(b)** PCR was used to validate the deletion of the *stuA* gene and complementation with the primers as indicated. Primer pairs: stuA-outside-F/HPH-R and HPH-F/stuA-outside-R were used to amplify the 5’ and 3’ flanking sequences, respectively. Primer pair of stuA-inside-F/stuA-inside-R was used to amplify the *stuA* fragment. (**c)** Southern blot analysis to examine the integrity of *stuA*. Genomic DNA purified from Δ*stuA*, Z7 and Δ*stuA*-C was digested with *Hind*III, electrophoresed in a 0.8% agarose gel, blotted to membrane, and hybridized with a *stuA* probe.

**Fig. S3 Site-directed mutagenesis analysis with the StuA protein**. **(a)** Four-day-old PDA cultures of the wild type strain Z7, Δ*stuA* mutant, Δ*stuA*-C^S67A^, Δ*stuA*-C^T110A^, Δ*stuA*-C^S370A^, Δ*stuA*-C^S411A^, and Δ*stuA*-C^T601A^ transformants, and complementation strain Δ*stuA*-C. **(b)** Detached Hongjv leaves inoculated with the same set of strains were examined for necrotic lesions symptoms at 3 dpi. Mycelial plugs of the mutants and wild-type strains were inoculated on the left and right sides of the main veins on the adaxial surface of the Hongjv leaves, respectively. **(c)** HPLC analysis of ACT toxin purified from culture filtrates of each strain. The peak representing ACT toxin is indicated by a black arrow. **(d)** Hyphae of transformants Δ*stuA*::StuA-GFP strain and the Δ*stuA*::StuA^T110A^-GFP were stained with 4',6-diamidino-2-phenylindole (DAPI) and examined by epifluorescence microscopy. Scale bar = 5 μm.

**Fig. S4 Verification of *pacC* mutants**. **(a)** Domain architecture of PacC deduced by SMART (http://smart.embl-heidelberg.de) showing three Cys2His2 zinc finger DNA-binding domains at the C terminal. **(b)** Phylogenetic analysis of PacC with fungal homologues, including *A. nidulans* (XP_660459), *A. niger* (XP_001399922), *P. digitatum* (AFS18474), *P. chrysogenum* (XP_002561889), *F. oxysporum* (EGU85857), *F. graminearum* (ADO60821), *M. oryzae* (XP_003713788), *B. cinerea* (XP_024551518), *S. cerevisiae* (NP_011836) and *C. albicans* (Q9UW14). The phylogenetic tree was reconstructed using a maximum likelihood (ML) method after alignment by the CLUSTALW algorithm using the MEGAX program. The horizontal bar indicates the relative distance in the phylogenetic tree. **(c)** Construction of the *pacC* RNAi vector pSilent-1-*pacC***. (d)** The relative expression level of *pacC* in different isolates containing pSilent-1-*pacC*, and the wild-type Z7, as determined by quantitative RT-PCR. (4) The relative expression level of the *pacC* in Z7, Δ*stuA*, Δ*stuA*-C, and Δ*stuA*-C^T110A^ strains, as determined by quantitative RT-PCR. Error bars represent standard deviations from three biological replicates. Different letters represent statistical significance according to the one-way ANOVA test (*p* < 0.05).

**Fig. S5 The relative area of necrotic lesions caused by *A. alternata* strains to inoculated leaves. (a)** Quantification of the necrotic leaf areas caused by mycelial plugs of Z7, Δ*stuA* and Δs*tuA*-C. **(b)** Quantification of the necrotic leaf areas induced by ACT crude extracts extracted from Z7, Δ*stuA*, and Δ*stuA*-C. **(b)** Quantification of the necrotic leaf areas induced by the mycelial plugs of Z7, Δ*stuA*, Δ*stuA*-C^S67A^, Δ*stuA*-C^T110A^, Δ*stuA*-C^S370A^, Δ*stuA*-C^S411A^, and Δ*stuA*-C^T601A^ and Δ*stuA*-C strains. The relative areas were calculated by image J.

**Table S1 Oligonucleotide primers used in this study**

**Table S2 Putative PKA phosphorylation sites of StuA**

**Table S3 Proteins putatively interacting with SOK2 in *S. cerevisiae***
