# Supplementary material for: The Alternaria alternata StuA transcription factor interacting with the pH-responsive regulator PacC for the biosynthesis of host-selective toxin and virulence in citrus
Source: Microbiol Spectr. 2023 Oct 9;11(6):e02335-23. doi: 10.1128/spectrum.02335-23 (PMC10715145; doi:10.1128/spectrum.02335-23)
Supplement: Table S2 — Putative PKA phosphorylation sites of AaStuA. [file spectrum.02335-23-s0008.pdf]

Sequence	Amino Acid	Position #	Score	Potential Kinase
AMVPSTLQL	Serine	67	0.556	PKA
KPRVTATLW	Threonine	110	0.653	PKA
SFDGSRPLY	Serine	370	0.548	PKA
PSRISDVPD	Serine	411	0.513	PKA
KRRKTMEGG	Threonine	601	0.565	PKA
